# Errors in Figure 4

**DOI:** 10.1001/jamanetworkopen.2026.40849

**Published:** 2026-09-28

In the Original Investigation titled “Intravenous Thrombolysis Beyond the Conventional Time Window for Acute Ischemic Stroke: A Systematic Review and Meta-Analysis”^1^ published August 6, 2026, there were errors in row headers of Figure 4, panels A and B. This article has been corrected.^1^
